# Training Data Extraction and Object Detection in Surveillance Scenario †

**DOI:** 10.3390/s20092689

**Published:** 2020-05-08

**Authors:** Artur Wilkowski, Maciej Stefańczyk, Włodzimierz Kasprzak

**Affiliations:** Institute of Control and Computation Engineering, Warsaw University of Technology, Nowowiejska 15/19, 00-665 Warszawa, Poland; maciej.stefanczyk@pw.edu.pl (M.S.); wlodzimierz.kasprzak@pw.edu.pl (W.K.)

**Keywords:** object detection, few shot learning, SVM, CNN, cascade classifier, video surveillance

## Abstract

Police and various security services use video analysis for securing public space, mass events, and when investigating criminal activity. Due to a huge amount of data supplied to surveillance systems, some automatic data processing is a necessity. In one typical scenario, an operator marks an object in an image frame and searches for all occurrences of the object in other frames or even image sequences. This problem is hard in general. Algorithms supporting this scenario must reconcile several seemingly contradicting factors: training and detection speed, detection reliability, and learning from small data sets. In the system proposed here, we use a two-stage detector. The first region proposal stage is based on a Cascade Classifier while the second classification stage is based either on a Support Vector Machines (SVMs) or Convolutional Neural Networks (CNNs). The proposed configuration ensures both speed and detection reliability. In addition to this, an object tracking and background-foreground separation algorithm is used, supported by the GrabCut algorithm and a sample synthesis procedure, in order to collect rich training data for the detector. Experiments show that the system is effective, useful, and applicable to practical surveillance tasks.

## 1. Introduction

Police and various security services use video analysis when investigating criminal activity. Long surveillance videos are increasingly searched by dedicated image analysis software to detect criminal events, to store them, and to initiate proper security actions. One of the prominent examples is the P-REACT project [1] (Petty cRiminality diminution through sEarch and Analysis in multi-source video Capturing and archiving plaTform). Solutions to automatic analysis of surveillance videos seem already to be mature enough, as the research community is recently also involved in significant benchmark initiatives [2,3]. The computer vision research focus is now shifted to the analysis of video data coming from handheld, body-worn, and dashboard cameras and on the integration of such analysis results with police- and public-databases.

In typical object detection scenarios, there are much data to learn from and a major objective is to use them effectively. In a security-oriented environment, the user interaction should be kept as simple as possible. The optimal solution would be marking only a single object in a selected image frame and initiating a search to find occurrences of similar objects in other frames of the processed sequence or different sequences. This imposes several constraints on the Machine Vision solution that need to be addressed.

First of all, the system should learn on-line or nearly on-line. The system must also perform per-frame detection quickly and provide approximate results in a short time, but also should be tuned in such a way, that no occurrence of the interesting object is skipped. Last but not least, the system must be able to learn from small data sets.

This paper is an extension of our conference paper [4] describing an effective and time-efficient algorithm for instance search and detection in images from handheld video cameras. The system described there uses a discriminant approach to differentiate the object from its foreground. To do so, a combined Haar–Cascade detector and Histogram of Oriented Gradients–Support Vector Machine (HOG-SVM) classifier are used. We argued that this provided a desirable trade-off between detection quality and training/detection times. Both the positive, as well as negative samples, are extracted only from training images.

The extended version presented here includes new system elements and experiments. In particular, the additions are as follows:

- introduction of a new foreground/background segmentation procedure,
- incorporation and evaluation of a CNN classifier in the detector framework,
- additional experiments covering new and previous features,
- extended state-of-the-art analysis.

**Figure 1:**
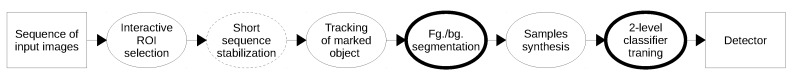
Structure of the training procedure.

The main components of the system that are affected by the additions were marked bold in Figure 1.

Comparable detector solutions based on CNNs provide excellent detection performance [5]. Such solutions, however, rely on off-line training and the training/detection speed is still a bottleneck for such systems. This effect is, to some extent, ameliorated by GPU utilization. Recent developments aim at the reduction of detection times by cascading CNNs [6] or by detecting salient regions first using fuzzy logic [7]. However, a significant reduction of training time is still an open area of research.

This work can be categorized as the few-shot learning system. The most popular approaches in this field focus on distance metric learning (which, on its own, has a long history [8]) with further data clustering using the $L_{2}$ metric. In recent years, new solutions based on deep learning emerged [9], with relational networks being the popular choice for few-shot learning [10]. A popular choice for the loss function, in that case, is triplet loss [11]. The application of distance metric learning for classification is straightforward. Metric is produced based on training samples, and the queries embed-dings are compared with the class representatives, with $L_{2}$ metric and/or *k*-Nearest Neighbor. This approach proved to be successful in multiple classification tasks [12,13,14,15].

Application of few-shot learning for detection is a harder problem, as usually the marked object in training set occupies only a small portion of the image. Hence, the training data set is heavily unbalanced. One of the possibilities is to transfer the knowledge from existing detectors [16]. Another alternative is to use semi-supervised learning, with few labelled samples and a larger set of unlabelled data, iteratively used in training [17]. A different approach is described in [18], where the imitations are used in the training process for a robot-grasping task. Finally, the direct application of distance metric learning was proposed in [19], where the InceptionV3 network is used as a backbone for metric learning.

Using the taxonomy proposed in [20] (data-, model-, or algorithm-based), our solution is data-based, with handcrafted rules for transforming the training dataset.

One contribution of the paper is the procedure of collecting as much realistic training data as possible, providing limited user interaction. Another contribution involves the proposition of a complete computer-aided video surveillance procedure and a thorough evaluation of the applicability of particular computer vision methods for specific stages of processing, having in mind the system requirements such as the ability to learn from short video sequences, performing quick learning and reaching fast detection time. The evaluation comprised both classic computer vision methods as well as contemporary CNN approaches.

Ideally, the modeling stage should be able to start from a single selection of a Region of Interest (ROI), and all additional examples should be obtained automatically. Such least-user-effort approaches were already discussed e.g., for semi-automatic video annotation and detection systems, such as [21,22]. In the cited method, however, the user may be asked to annotate video several times (to decide about samples lying on decision boundary), which is not necessarily acceptable for all end users. An example of another successful detector that works on a single selection is given in [23]. The detector operates on sparse image representation (collection of Scale Invariant Feature Transform (SIFT) descriptors), so it is very time efficient. Our initial experiments have shown that descriptor-based approaches work the best for highly textured and fairly complex objects that occupy a large part of the image, which is not always true in the surveillance scenarios.

The procedure of collecting training data, given in this paper, combines object tracking and background subtraction methods for semi-supervised collection of training windows and their foreground masks. This step is supported by the GrabCut algorithm [24], with the possibility of smoothly mixing the results of both. The samples collected during tracking are further synthetically generalized (augmented) to enrich the training set. Scenarios, where tracking results are utilized for the collection of detector’s training data, were already covered in literature, especially regarding tracking, with prominent examples [25,26] or more recent CNN approaches [27,28]. In such approaches, the exact foreground–background separation (which is crucial for effective synthesis of samples) is often neglected, since the algorithms typically have enough frames to collect rich training data.

The proposed methods were evaluated on a corpus of surveillance videos and proved that its efficiency is good enough to be effective in supporting a user (police officer or security official) in their everyday working tasks. The proposed two-level detector architecture was evaluated using alternative methods for feature calculation, namely Histogram of Oriented Gradients (HOG) features and VGGNet-based deep features. In the former case the SVM classifier was used, in the latter, the neural network classification layers were consistently utilized. For final evaluation we compared the accuracy of our proposed system with the state-of-the-art Faster-RCNN [29] detector trained on the same data.

The paper is organized as follows: in Section 2 there is given a technical background and methodology used in our system, Section 3 provides experimental results, and Section 4 contains conclusions. For the reader’s convenience, a short dictionary of abbreviations used in the paper is presented at the end.

## 2. Methods

### 2.1. Detector Overview

In the system described in this paper, we utilize a classic detection framework, where a sliding window with varying sizes is moved over each frame and, for each location, the selected image part is evaluated against information gathered from training samples. A crucial part of the detector is formed by a classifier (SVM or NN), which is responsible for the evaluation of each selected image part. A pure classifier, when applied to hundreds of thousands of candidate areas, would be too slow to learn and detect. In our scenario, a pre-classification step utilizing Haar-like feature-based cascade classifier is applied to limit the number of candidate windows to several hundred. We claim that this simple structure combines a good detection rate with acceptable detection speed (about ten full-HD frames per second on modest computer) as well as acceptable training speed in typical scenarios (less than a few minutes per pattern).

In essence, the two-stage detector architecture resembles some significant modern CNN approaches, where the detection is divided into the region proposal part and the region recognition part [29]. In our approach, region proposal is performed by the cascade classifier, and the (SVM/NN) classifier does the final classification. Both methods offer reasonable training and detection speeds required for this application.

In our scenario, sources of data are naturally sparse. Depending on user decision, the detector can be trained either on one or a short sequence of training images. Therefore a critical part of our system is a set of tools aiding the user in an effortless collection of training examples from short image sequences as well as methods for artificial synthesis and generalization of training samples to provide the detector with the training data as rich as possible. These tools and methods are discussed in subsequent sections. The overall structure of the training procedure is given in Figure 1.

The first stage of processing is and interactive collection of training samples (*Interactive ROI selection*). The user marks an object in the image and initializes the tracking procedure to collect more training data for the selected object (*Tracking of marked object*). In case of un-stabilized images (e.g., from hand-held camera) additional stabilization step can be applied (*Short sequence stabilization*). The tracking is open to user intervention e.g., correction of the ROI in a single frame or frames.

The next step of the procedure is foreground–background segmentation (*Fg./Bg. segmentation*), which helps to recover the precise outline of the object from the rectangular ROI basing on motion and color information. After this comes *Samples synthesis*, here, the collected training samples are jittered in different ways to enrich the training set. Both steps enable the operator to correct algorithm parameters with visual feedback. The last step of detector generation is the training of a 2-level classifier (*2-level classifier training*).

### 2.2. Collection of Positive Training Samples

Although for some patterns (which include e.g., flat patterns) good detection results can be obtained using only one selected sample that is further generalized and synthesized into a set with larger variability, in most cases detection results highly depend on size and diversity of input training set. In the scenario discussed in this paper, these properties of the training set can (at least partially) be achieved by collecting samples from a short sequence of input images. Our scenario is organized as follows: (1) a user selects an object of interest using a rectangular area, (2) the application tracks the object in subsequent frames of the sequence (with optional manual reinitialization), (3) object foreground masks are established using motion information and image region properties.

#### 2.2.1. Object Tracking and Foreground–Background Separation Using Motion Information

For tracking of rectangular area an optimized version of Circulant Structure of Kernels (CSK) tracker [30] that utilizes color-names features [31] is used. As a result of the tracking procedure, we obtain a sequence of rectangular areas that encompass the object of interest in subsequent frames. In most cases both object foreground, as well as background, will be present in the tracked rectangle. However, if the object is moving against a moderately static background, we can exploit motion information to effectively separate object foreground from background by background subtraction.

Let the tracking results be described by a sequence of rectangular areas $\left\{R^{1},\ldots ,R^{T}\right\}$ and let us denote coordinates of pixel *i* as $p_{i}$, color attributes for pixel *i* at time *t* as $c_{i}^{t}$, and a mean of color attributes in the background as:

(1) $${\bar{c}}_{i}=\frac{1}{n_{i}}\sum_{t:p_{i}\notin R^{t}}c_{i}^{t}$$

where averaging factor $n_{i}$ is the number of frames where tracking window does not contain pixel *i* and can be computed as $n_{i}=\left|\left\{t:p_{i}\notin R^{t}\right\}\right|$.

Now we can specify a background training sequence for each pixel $\left\{{\hat{c}}_{i}^{t}\right\}$

(2) $${\hat{c}}_{i}^{t}=\left\{\begin{array}{cc} c_{i}^{t} & \mathrm{if}\;p_{i}\notin R^{t}\\ {\bar{c}}_{i} & \mathrm{if}\;p_{i}\in R^{t} \end{array}\right.$$

Following the rule above, only pixels that at given time-step do not belong to the tracked area contribute to the background model computed for the image. Each pixel that always belongs to the tracked area is conservatively treated as a foreground as we are unable to establish a background model for these areas.

The background model adopted here follows algorithms from [32]. In this method, scene color is represented independently for all pixels. The color for each pixel (both from the background and foreground BG+FG) given the training sequence $C_{T}$, is modeled as:

(3) $$p\left(c_{i}|C_{T},BG+FG\right)=\sum_{m=1}^{M}{\hat{\pi }}_{m}N\left(c_{i};{\hat{\mu }}_{m},{\hat{\sigma }}_{m}\right)$$

where ${\hat{\mu }}_{m},{\hat{\sigma }}_{m}$ are estimated means and standard deviation of color mixture components, ${\hat{\pi }}_{m}$ are mixing coefficients, *M* is the total number of mixtures, and $N(c_{i};{\hat{\mu }}_{m},{\hat{\sigma }}_{m})$ denotes Gaussian density function evaluated in $c_{i}$.

In the algorithm a (generally correct) assumption is made that background pixels, as appearing most often, will dominate the mixture. Therefore the background model (BG) is built from the selected number of largest clusters in the color mixture:

(4) $$p\left(c_{i}|C_{T},BG\right)=\sum_{m=1}^{B}{\hat{\pi }}_{m}N\left(c_{i};{\hat{\mu }}_{m},{\hat{\sigma }}_{m}\right)$$

where *B* is the selected number of background components. The pixel is decided to belong to the background when

(5) $$p\left(c_{i}|C_{T},BG\right)>c_{thr}$$

**Figure 2:**
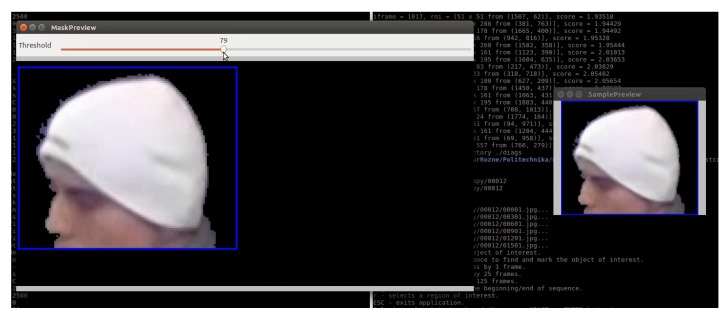
Results of automatic foreground–background separation.

Threshold $c_{thr}$ can be interactively adjusted by the user. Exact algorithms for updating mixture parameters are given in [32]. Sample result of background subtraction procedure is given in Figure 2.

Some modern developments in foreground–background separation using the Robust Principal Component Analysis (RPCA) approach were proposed e.g., in [33,34]. They are founded on extensive optimization in 3D spatio-temporal volumes and offer excellent accuracy at the expense of some processing speed. Since our system relies on the interaction between the human operator and computer, the processing speed is very important, so purely local methods seem to be currently the best choice. However, this topic will be investigated in the future versions of the system since some ideas from [33,34] are likely to be complementary to our developments presented in Section 2.2.3.

#### 2.2.2. GrabCut Algorithm

GrabCut [24] is a widely acclaimed method for (semi)automatic foreground/background segmentation. The method takes into account several properties or image regions: color distribution, coherence of regions, contrast between regions. These factors are described in the form of image-wide energy function to optimize, that assumes the following form:

$$E\left(\underline{\alpha },\underline{\theta },z\right)=U\left(\underline{\alpha },\underline{\theta },z\right)+V\left(\alpha ,z\right)$$

where $\alpha_{n}$ describes segmentation information for pixel *n* (can be binary), $\underline{\theta }$ is the set of parameters of the Gaussian Mixture Model representing color distribution in background and foreground and z are observed image pixels. Estimated parameters are underlined. The energy term *U* describes, how well current estimation of foreground and background pixels matches the assumed color distribution of foreground and background, while the term *V* evaluates spatial consistency of regions by penalizing discontinuities (except for the areas of high contrast).

The best configuration of parameters is the one minimizing the term $E(\underline{\alpha },\underline{\theta },z)$. The components are designed in a way that the energy term can be minimized using an effective graph-cut algorithm. The optimization algorithm is iterative and switches between (re)estimation of region color distribution and (re)estimation of segmentation.

The input of the algorithm is defined in [24] as a trimap $\left\{T_{B},T_{U},T_{F}\right\}$. $T_{B}$ stands for sure background, $T_{F}$ stands for sure foreground, and $T_{U}$ is an unknown area (to estimate). $T_{B}$ and $T_{F}$ are fixed and cannot change during the algorithm. Typical initialization is to set $T_{B}$ to the area outside of object ROI, set $T_{F}$ to ∅, and $T_{U}$ is the remaining part of the image. In the first iteration, all pixels from $T_{B}$ are initialized as background and all pixels from the unknown area $T_{U}$ as foreground (which is subject to change). Implementations like [35], however, allow to specify the additional areas within $T_{U}$: the likely foreground ${\hat{T}}_{F}$ and likely background ${\hat{T}}_{B}$ as a convenient starting point for optimization.

#### 2.2.3. Object Tracking and Foreground–Background Separation Using Hybrid Motion Information and GrabCut

While pure object motion information is sufficient to perform foreground–background segmentation in most cases, it fails altogether for static objects. In addition, the precision of such an approach varies from case to case and strongly depends on the manual threshold selection for background subtraction $c_{thr}$. Therefore we propose a modified procedure of fine-tuning results of background subtraction using GrabCut.

1. The object is tracked and its foreground mask is obtained using methods from Section 2.2.1.
2. For each frame the current foreground–background segmentation results are used to initialize a GrabCut trimap, specifically:
  - the foreground region is used to initialize the G-C $T_{F}$, with an exception for the area for which no background model could be reliably established (areas that belong to each collected tracking ROI),
  - the G-C $T_{B}$ is initialized outside the tracked ROI border, to provide enough pixels for background estimation the tracking area is scaled uniformly by 50%,
  - the remaining area of ROI becomes the $T_{U}$.

We found our solution somewhat similar to the one proposed quite recently in [36]. However, in the cited approach the trimap is initialized differently. The G-C $T_{U}$ area is limited only to the area of the morphological gradient (difference between dilation and erosion) of the foreground area established by background subtraction. In our solution we safely assume that $T_{B}$ is always outside the tracked ROI, so there is no risk to incorporate the foreground object in $T_{B}$. Additionally, in contrast to our approach, in [36] the background subtraction is not discussed within the tracking context.

The solution proposed here is parametrized by a single background subtraction threshold $c_{thr}$ and provides a smooth user experience when transiting between different threshold values. By specifying very low thresholds, the user selects a foreground mask covering the whole tracked ROI. For larger thresholds, we obtain the results of G-C algorithm with foreground constrained to be at least the mask generated by background subtractor. For very large, extreme values of $c_{thr}$, the foreground seeds from the background subtractor become small, and the method converges to the output of the vanilla G-C algorithm for static images.

This is interesting to note that for static ROIs we use exactly the same procedure. For such ROIs, the area of uncertain background model (the area where no background model could be reliably established) is very large and covers the whole ROI. In such a situation, the entire ROI area is simply a subject to the classic G-C algorithm. Note also, that using only methods from Section 2.2.1 the whole ROI area would be inevitably labelled as foreground.

#### 2.2.4. Image Stabilization in a Short Sequence

The foreground–background segmentation procedure works best when the stable camera position is available (or image sequence is stabilized before segmentation). The system proposed here uses a stabilization procedure basing on matching of SURF features [37] and computation of homography transformation between pairs of images. The stabilization works on short subsequences of the original sequence. The first frame to stabilize is the one used for marking the initial region of interest. The procedure then aligns all subsequent frames to the first frame by evaluating homography relating two images. In order to do so, matching methods from [38] and the Least Median of Squares principle [39] are utilized. To increase stabilization efficiency, GPU-accelerated procedures for keypoints/descriptors extraction and matching from OpenCV library are utilized [35].

### 2.3. Collection of Negative Training Samples

**Figure 3:**
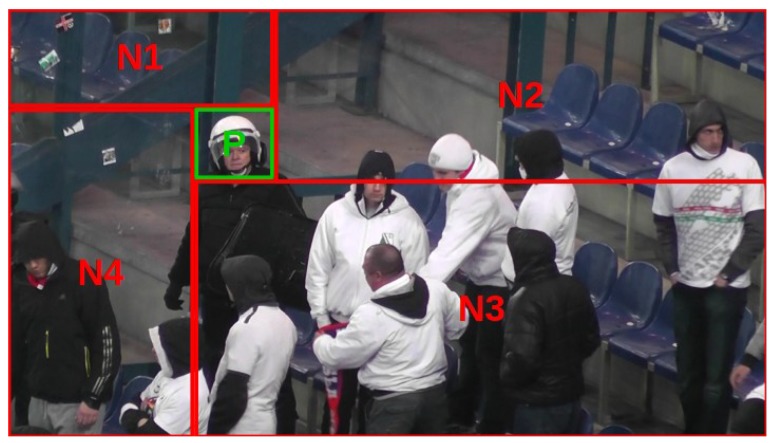
Division into positive (P) and negative (N1–N4) examples.

Negative samples that are used in detector training are extracted from the same sequence images that positive samples originated from. For each training image, one fragment is used to extract a positive sample, while the remaining part of the image is divided into at most four sources of negative samples, as given in Figure 3. Thus, an assumption is made that these remaining parts of the training sequence images do not contain positive samples. This assumption is not always valid, but may be strengthened by asking a user to mark all positive examples in the training sequence.

### 2.4. Positive Samples Generalization and Synthesis

#### 2.4.1. Geometric Generalization

In this step, 3D rotations are applied to collected pattern images and their masks. It is assumed that patterns are planar, so this generalization method can be useful only to some extent for non-planar objects. The rotation effect is obtained by applying a homography transformation, imitating application of three rotation matrices $R_{x}\left(\alpha \right)$, $R_{y}\left(\beta \right)$, $R_{z}\left(\gamma \right)$ to a 3D object. The matrices correspond to rotations around *x*, *y*, and *z* axes correspondingly. 3D rotation matrices are defined classically:

(6) $$\begin{array}{c} \begin{array}{c} R_{x}\left(\theta \right)=\left(\begin{array}{ccc} 1 & 0 & 0\\ 0 & \cos\theta & -\sin\theta \\ 0 & \sin\theta & \cos\theta \end{array}\right)\\ R_{y}\left(\theta \right)=\left(\begin{array}{ccc} \cos\theta & 0 & \sin\theta \\ 0 & 1 & 0\\ -\sin\theta & 0 & \cos\theta \end{array}\right)\\ R_{z}\left(\theta \right)=\left(\begin{array}{ccc} \cos\theta & -\sin\theta & 0\\ \sin\theta & \cos\theta & 0\\ 0 & 0 & 1 \end{array}\right) \end{array} \end{array}$$

To compute the transformation, first a homography matrix is computed using formula:

(7) $$H=R-\frac{tn^{T}}{d}$$

where n is a vector normal to the pattern plane (we set it to $n={\left(0,0,1\right)}^{T}$), *d* is the distance from the virtual camera to the pattern (we set it arbitrarily to d=1, since it only scales ’real-world’ units of measurement) and *R* is the 3D rotation matrix that is decomposed as:

(8) $$R={\left(R_{x}\left(\alpha \right)\cdot R_{y}\left(\beta \right)\cdot R_{z}\left(\gamma \right)\right)}^{-1}$$

In order for the image center (having world coordinates $C={\left(0,0,d\right)}^{T}$) to remain intact during transformation we define ’correcting’ translation vector as:

(9) t=−RC+C

Then we can specify artificial camera matrices as $K_{1}$ and $K_{2}$

(10) $$K_{1}=\left(\begin{array}{ccc} f & 0 & c_{in}^{x}\\ 0 & f & c_{in}^{y}\\ 0 & 0 & 1 \end{array}\right),\;K_{2}=\left(\begin{array}{ccc} f & 0 & c_{out}^{x}\\ 0 & f & c_{out}^{y}\\ 0 & 0 & 1 \end{array}\right)$$

where ${\left(c_{in}^{x},c_{in}^{y}\right)}^{T}$ and ${\left(c_{out}^{x},c_{out}^{y}\right)}^{T}$ are pixel coordinates of input and output image correspondingly, while *f* is the artificial camera focal length given in pixels. In this application we set *f* to be $f_{mul}$ times larger input image dimension. Multiplier $f_{mul}$ decides about the virtual distance of our virtual camera to the object. Smaller values introduce larger perspective distortions of the transformation, larger values introduce smaller distortions. We arbitrarily set $f_{mul}$ to 10 implying only slight perspective distortions.

The final homography transformation applied to the pixels of the input image is given by

(11) $$P=K_{2}HK_{1}^{-1}$$

Rotation angles α, β, and γ are selected randomly from the uniform distribution (denoted here as U). The amount of rotation around axes *y* is twice times the amount of rotation around remaining axes to better reflect dominant rotations in human movement

$$\begin{array}{cc} \alpha & ∼U\left(-1,1\right)\cdot \delta_{max}\cdot 0.5,\\ \beta & ∼U\left(-1,1\right)\cdot \delta_{max},\\ \gamma & ∼U\left(-1,1\right)\cdot \delta_{max}.\cdot 0.5 \end{array}$$

and $\delta_{max}$ is the parameter specifying the maximum extent of allowed rotation.

#### 2.4.2. Intensity and Contrast Synthesis

In the proposed approach image intensity and contrast synthesis is applied in addition to geometric transformations. It is especially important for Haar-like features that lack intensity normalization.

First, intensity values of pixels $I_{in}$ are retrieved from RGB image by extracting V component from HSV representation of the image and setting $I_{in}=V$. The intensity and contrast adjustment affects only V channel. After adjustment, the RGB image is reconstructed from HSV′ where $V^{\prime }=I_{out}$.

For adjustment, a simple linear formula is used. For each pixel gray value $I_{in}$ we have

(12) $$I_{out}=a*I_{in}+b$$

where

(13) $$a=1+c_{dev},\;b=I_{dev}-\mu_{I}\cdot c_{dev}$$

where $\mu_{I}$ is the average intensity of the sample. Contrast deviation $c_{dev}$ as well as intensity deviation $I_{dev}$ are sampled from the uniform distribution $c_{dev}∼U\left(-1,1\right)\cdot c_{max}$ and $I_{dev}∼U\left(-1,1\right)\cdot I_{max}$. $c_{max}$ is a parameter denoting the maximum allowed contrast change and $I_{max}$ is a parameter denoting the maximum allowed intensity change. Changes in contrast preserve mean intensity of an image. After application of the formula its results are appropriately saturated.

#### 2.4.3. Application of Blur

Training and test samples may differ in terms of quality of image details due to different factors such as deficiencies of optics used, motion blur or distance. In our case, we apply a simple Gaussian filter to simulate natural blur effects

(14) $$\sigma =U\left(0,1\right)\cdot \sigma_{max}\cdot \min\left(I_{width},I_{height}\right)$$

where $I_{width}$ and $I_{height}$ are image sample sizes and $\sigma_{max}$ controls the maximum size of the Gaussian kernel.

#### 2.4.4. Merging with the Background

Generalized training images are superimposed on background samples extracted from negative examples of size ranging from about 0.25 to 4 times the positive sample size. Gray-level masks are used for the seamless incorporation of positive samples into background images.

### 2.5. Detector Training

The detector training procedure is divided into two steps. In the first step, the cascade classifier using HAAR-like features is trained. The classifier is trained on training samples resampled to a fixed size of 24 × 24 pixels. In our scenario, for each cascade stage, 300 positive samples and 100 negative samples are utilized. The minimum true positive rate for each cascade level is set to 0.995, and the maximum false positive rate is set to 0.5. The classifier is trained for a maximum of 15 stages or until reaching ≈0.00003 FPR. The expected TPR is at least $0.995^{15}\approx 0.93$. By using these settings, up to about 1000 detections are generated for each Full-HD test image.

### 2.6. Detector Training Using HOG+SVM

During the second stage of training an SVM classifier is trained to handle samples that passed the first cascade classification. For most experiments, the SVM classifier is trained on 300 positive and 300 negative samples or 600 and 600 samples accordingly. The SVM classifier uses the Gaussian RBF kernel.

(15) $$K\left(x,y\right)=\exp(-\gamma ||x-y|{|}^{2})$$

The Gaussian kernel size γ and SVM regularization parameter *C* are adjusted using automatic cross-validation procedure performed on the training data. For SVM classification Histogram of Oriented Gradients features [40] are extracted. There were used 2 resolutions of training images: 24 × 24 and 32 × 32. For each sample a 9-element histogram in 4 × 4 cells is created with 16 × 16 histogram normalization window overlapping by 8 pixels, thus giving 4∗16∗9=576 HOG features in total in 24 × 24 case and 9∗16∗9=1296 in 32 × 32 case.

Negative samples are extracted from the Cascade Classifier decision boundary (containing samples that were positively verified by CC but still negative) if possible. If not, image fragments used as background images for positive samples or (as the last resort) other randomly selected samples are used. In all experiments OpenCV 3.1 [35] Cascade Classifier and SVM implementation are utilized.

Given our test data, the number of resulting support vectors in the SVM classifier varies between 200 and 400 for 24 × 24 case but can be twice as large for 32 × 32 case. Let us review one specific configuration: ’hat’ pattern trained on 55 24 × 24 images with masks and pattern generalization settings $\sigma_{max}=c_{max}=0$, $\delta_{max}=0.7$, $I_{max}=50$. After SVM metaparameter optimization we obtain SVM regularization parameter C=2.5, RBF kernel size γ=0.5, and the number of support vectors 233.

### 2.7. Detector Training Using Tuned VGG16 Network

The number of features processed by our detector is limited to HOG and Haar-like features. There exist quite a few other solutions that use a much richer set of features for object detection e.g., [41], however, at the expense of increased computational complexity, which is an important practical concern of our system. As an alternative to evaluating complex sets of hand-crafted features, we decided to resort to the state-of-the-art methods automatic feature computation based on Convolutional Neural Networks.

Therefore in addition to the classifier described in Section 2.6, we evaluate a VGG16 network [42] trained on the ImageNet dataset [43] tuned to our problem using transfer-learning principle [44]. Transfer learning is a common technique to overcome the problem of the availability of training data. In case of a limited access to the training data for the given problem, one can use an existing network trained on another dataset and subsequently fine-tune (retrain) this network to accommodate the specific problem samples. Since usually the original network was trained on millions of examples and hundreds of classes, it is capable of extracting robust and usually quite universal features. Thus, the new network can benefit from pre-trained feature layers and train only classification layers.

**Figure 4:**
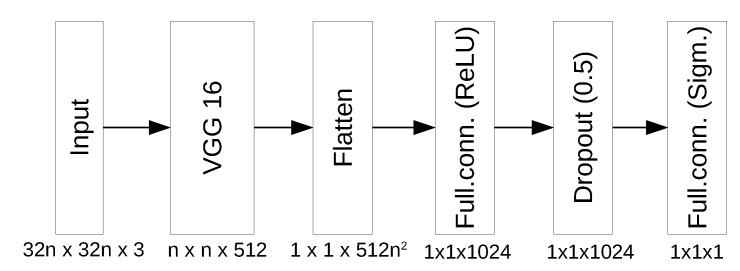
Neural Network Structure, n∈{1,2,4,7}.

For our task, we utilize a convolutional layer of VGG16 network (which is obtained from the original network by removing fully-connected layers). The network is augmented with two fully connected layers (1024 neurons with ReLu activation and a single neuron with sigmoid activation correspondingly) and one dropout layer (with dropout rate 0.5) to avoid overfitting. The VGG16 network was originally trained on a sample image of 224 × 224, however, the convolutional part would accept any multiplicity of 32 for image width and height. In this paper, we verify classifier performance for input image sizes 32 × 32, 64 × 64, 128 × 128, and finally 224 × 224 pixels. The total number of parameters in fully connected layers ranges from 522 337 to 25 692 161 depending on input image size. The overall network structure is given in Figure 4.

The selected loss function is a binary cross-entropy for binary classification. During training, all the convolutional layers are frozen and only the parameters of fully connected layers are adapted.

### 2.8. Detection and Post-Processing

During the detection phase, each test image is first processed by the cascade classifier, typically returning several hundreds of candidate areas. After this, each candidate area is examined by the classifier and a score is assigned to each detection. In case of the SVM, the score is computed as the signed distance from the separating plane in support vector space with the lowest negative scores treated as best matches and high positive scores as worst matches. For the VGG16-based neural network, the output of the sigmoid function is negated and used as the score.

For each image, only the best score area is considered for further processing. Frames from the test sequence are sampled and processed with increasing density (first, last, and middle frame for a start, and then intermittent frames), to quickly produce some results for the user to review (non-minima suppression is used to reduce clutter)

## 3. Experiments

### 3.1. Experimental Setup

In order to evaluate the detector performance the following tools are utilized.

- ROC curve: Receiver Operator Characteristic curve is the curve relating the True Positive Rate (TPR) and the False Positive Rate (FPR). The True Positive Rate (TPR) is defined as the number of correctly detected examples (TP) by the total number of positive occurrences (P), the False Positive Rate (FPR) is defined as the number of incorrectly detected examples (FP) by the total number of negative samples (N). The ROC curve gives comparable results even for the imbalanced datasets.
- PR curve: Precision-Recall curve is the curve relating Precision (the number of truly positive samples among all positive detections) and the Recall (another name for True Positive Rate). The PR curve is useful for establishing how many good hits can be expected among those best ranked by the detector.
- AUC: Area Under Curve is a useful single measure to summarize ROC curve, computed by the integration of TPR over FPR
- AVGPR: Average Precision-Recall is another a useful measure to summarize PR curve, computed by integration of the Precision over Recall
- EER: Equal Error Rate denotes a line on ROC plot where TPR + FPR = 1 or a point on ROC curve meeting this requirement

In the subsequent sections, each ROC and PR curve was plotted basing on a single run of the detector, whereas the majority of tables included in the subsequent sections contain aggregated data from multiple runs of the algorithm to compensate the stochastic nature of Machine Learning algorithms used. Computational performance experiments were performed on AMD Ryzen 5 2600X processor (32 GB of memory) and GeForce RTX 2070 GPU (8 GB of memory) unless stated otherwise.

### 3.2. Preliminary Experiments

**Figure 5:**
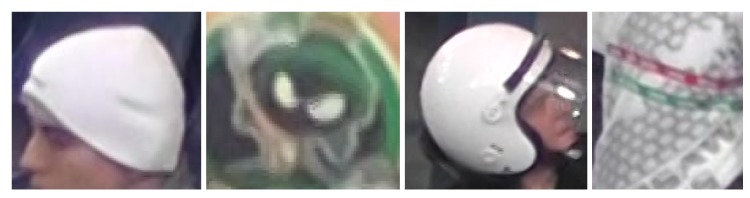
Example training samples of hat, logo, helmet, and shirt.

**Figure 6:**
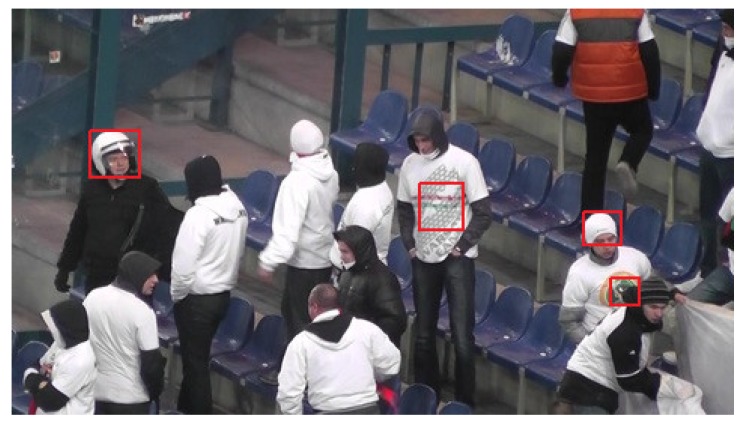
Frame with marked hat, logo, helmet, and shirt samples.

**Figure 7:**
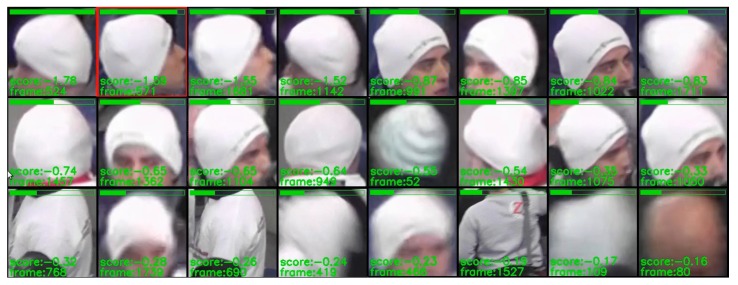
Detection results filtered by minimum distance (25 frames) between hits.

During the first stage of experiments there was selected a single test sequence ’00012’ with 1776 Full-HD frames. Using this sequence, various parameter configurations were evaluated in order to assess the basic properties of the solution proposed. Basing on these experiments, some answers can be given regarding problems such as the impact of utilization of a two-layer detector on detection results and detection/training speed, the impact of the method of selection of training samples on detection accuracy or influence of values of image synthesis parameters on overall quality. Above questions will be discussed in the following paragraphs. During the first three experiments, one sample pattern ’hat’ was utilized, and in the last experiment three other patterns: ’logo’, ’helmet’, and ’shirt’ were introduced. Examples of training samples are given in Figure 5, and samples marked in the full-frame image are given in Figure 6. Filtered detection results for one test sequence presented in the form of a simple GUI are given in Figure 7.

#### 3.2.1. Two-Layer Detector

**Figure 8:**
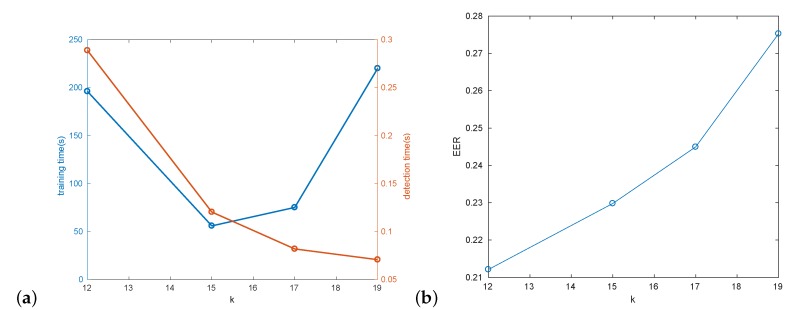
Impact of different number of cascades (*k*): (**a**) training/detection time; (**b**) hat detection EER.

In the first experiment a trade-off between the detection and training speed for different number of expected cascade stages *k* was evaluated (Figure 8a). In this experiment HOG+SVM second stage classifier was used. Identical parameters were used for all *k* values, except for the number of the SVM training samples. For k<15, a larger number of 900 positive and negative samples were used. For k≥15, the default of 300 positive and negative samples were utilized. This was used to balance the total number of training samples consumed by the detector (for smaller *k* the task of SVM is harder, since more negative samples pass the first stage of detection).

**Figure 9:**
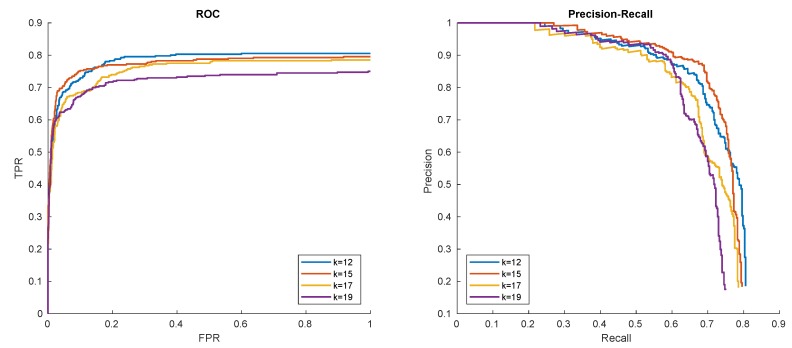
Hat in ’00012’ detection results with respect to number of the requested cascade stages.

The experiment shows, that for low *k* training time is dominated by SVM training, and for large *k* cascade training dominates. A good compromise for our data can be obtained for value of k=15. Larger *k* obviously means faster detection (Figure 8a), but also slightly worse detection results (Figure 8b), likely due to utilization of more robust HOG features in the second stage. This statement is also generally supported by the shape of corresponding ROC curves in Figure 9. These preliminary performance experiments were performed on Intel Core i5 computer.

#### 3.2.2. Collection of Training Samples

**Figure 10:**
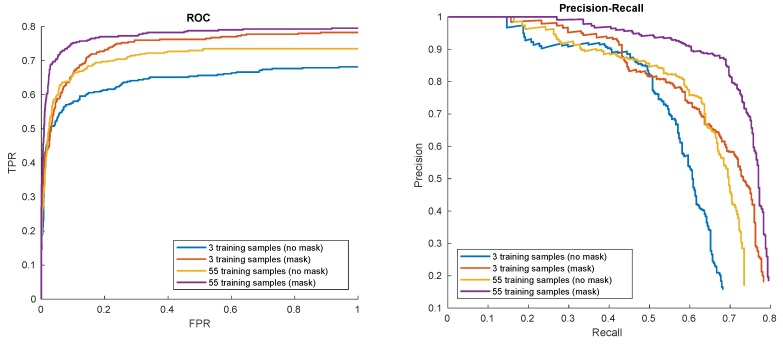
Hat in ’00012’ detection results for different training data collection methods.

In the next experiments, detector performance for different training data collection methods was evaluated. In the first place the data samples were collected using the automatic tracking and foreground–background separation methods given in this paper. In the process 55 data samples of hat pattern were collected, together with their automatically generated masks (using methods from Section 2.2.1). The data consisted of images of a white hat on top of a head, while the head was making full 180 degrees rotation around central axis. For comparison, a short sequence of training samples representing only 3 extreme head positions (*en-face* and two profiles) was utilized. For both sequences either appropriate foreground–background masks or no masks were used given 4 different combinations of settings. The detection results are given in Figure 10.

Not surprisingly, the richest possible data source (55 frames with generated masks) gives the best results. It is valuable to note that for our data, applying both object tracking and automatic mask generation is substantial to get optimal results.

#### 3.2.3. Application of the GrabCut Algorithm

In this paragraph, we evaluate the hybrid method for foreground/background segmentation utilizing background subtractor and GrabCut that was described in Section 2.2.3. It is evident that for single training images, the proposed segmentation method is reduced to pure GrabCut, and as such GrabCut becomes the only segmentation option, which should be beneficial in most cases (see: discussion on masks in the previous paragraph). However, the more interesting question is if GrabCut can effectively cooperate with background subtractor and enhance its results. Intuitively, it should be the case since both methods operate on different principles.

**Figure 11:**
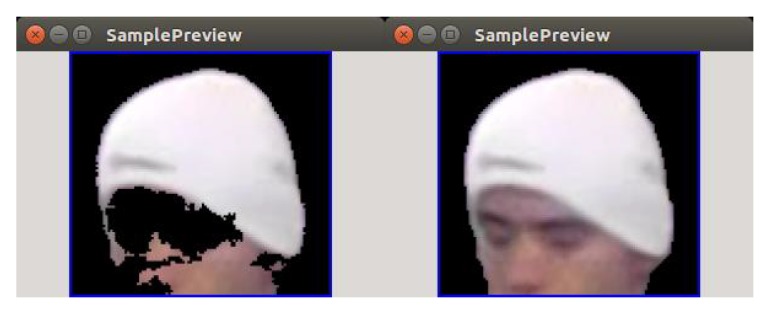
Foreground/background segmentation results using only background subtraction (on the left) and background subtraction + GrabCut (on the right) for background cut-off threshold $c_{thr}=600$.

**Figure 12:**
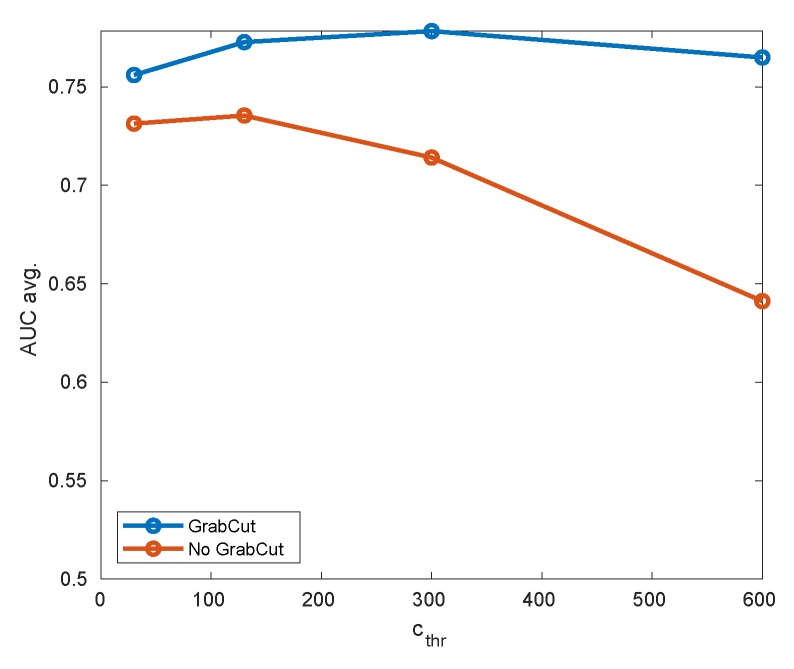
Comparison of detector accuracy with GrabCut turned on or off for different thresholds of background cut-off (pattern hat).

Taking a quick glance at Figure 11 shows us that this is actually the case, so the GrabCut building on the initial foreground/background segmentation can effectively smooth out imperfections in the initial estimation. In order to evaluate it quantitatively, we propose an experiment where the detection results are evaluated by a GrabCut module turned on and off for different values of background cut-off threshold $c_{thr}$). The results are summarized in the Table 1 and Table 2, and Figure 12.

The results can be summarized two-fold. Firstly, the compound algorithm (mixing background subtraction and GrabCut) offers better accuracy for all thresholds analyzed, especially for larger background cut-off thresholds. However, since GrabCut results must always contain the area initialized by B-S, this does not need to hold for extremely small thresholds (where the initial area is already very large).

Secondly, the performance of the pure background subtractor strongly depends on the cut-off threshold, while GrabCut seems to loosen this correlation. This may enable (in the future version of the system) to remove the threshold $c_{thr}$ altogether and to fix some reasonable default.

Finally we decided to evaluate the performance of 3 methods: pure background subtractor, the mixture of a background subtractor with GrabCut, and a successful contemporary tracker and mask generator [45]. The latter method relies on Siamese Network (a popular method for tracking applications), extended with the ability to generate binary foreground/background masks for the tracked object. In our experiments we used a network trained on a DAVIS dataset [46]. Comparison of three methods is given in Table 3.

Although the results are not fully comparable (e.g., we had to approximate more complex ROI returned by the SiamMask algorithm with a rectangular ROI for further utilization in training), we can observe that the performance of SiamMask is somewhat worse than the performance of the other two methods. Although the tracking process is fine, it can be observed that the generated mask does not correspond well to ground truth data. This effect could be easily attributed to a natural bias of the method towards specific classes of objects that it was trained on. However, if the method was trained on the data from a domain similar to our test data, the results could be much better. This is an open area for further research.

#### 3.2.4. Synthetic Generalization of Training Data

**Figure 13:**
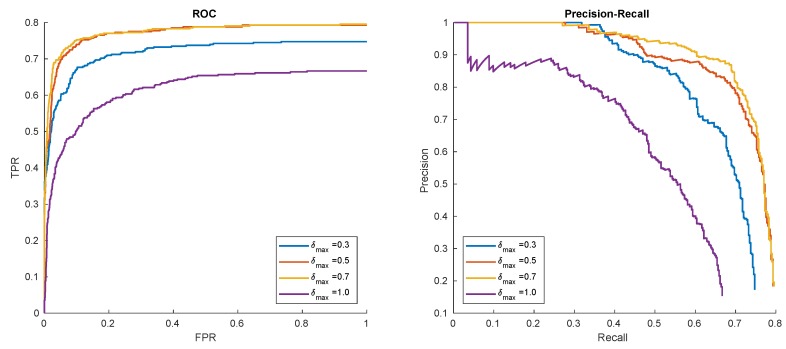
Hat in ’00012’ detection results for different levels of geometric synthesis.

**Figure 14:**
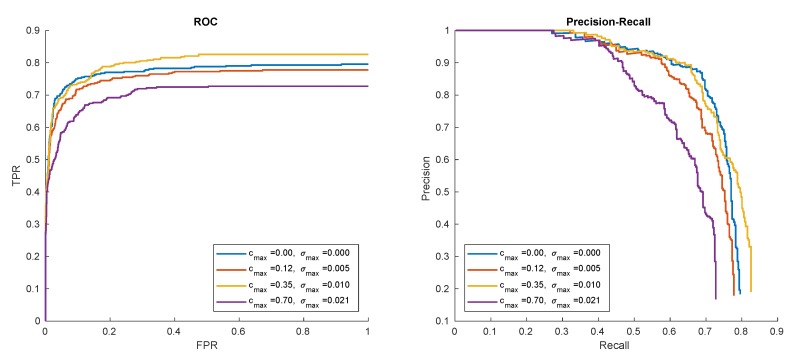
Hat in ’00012’ detection results for different contrast and sharpness synthesis levels.

In these experiments, different measures and intensity of synthetic samples augmentation were evaluated. The results are given in Figure 13 and Figure 14. The results show that moderate geometric, as well as contrast and sharpness generalization, provides the best results. However, the selection of appropriate parameters is object and sequence-specific. It may be observed that near-flat surfaces (like logo) benefits from aggressive geometric distortions (i.e., larger rotation angles). In addition, the reduction of sharpness proved to work best for computer-graphics-generated patterns.

#### 3.2.5. Selection of Training Data Size

**Figure 15:**
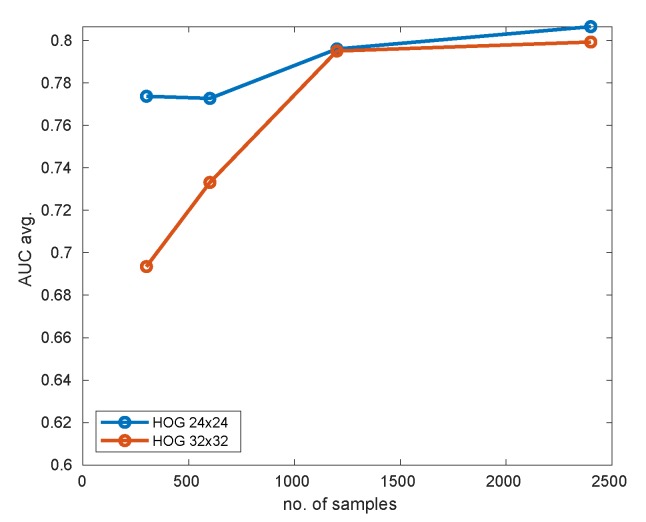
Comparison of detector performance using 24 × 24 and 32 × 32 HOG features for different sizes of the training set and hat pattern.

The selection of the appropriate size of the training dataset is crucial for detection accuracy as well as time performance. It applies especially to the 2-nd stage of detection since this is the layer that performs the final evaluation of samples. To tune our system with this respect, we performed an analysis using one pattern: hat. There was evaluated a HOG+SVM classifier. The input data was augmented basing on data resulting from foreground–background segmentation from Section 2.2.3 ($c_{thr}=130)$. The evaluated input image size were 24 × 24 and 32 × 32. The tested number of training images was 300, 600, 1200, and 2400. In all cases the SVM hyperparameters were trained using a 10-fold cross-validation procedure. The results obtained are given in the Table 4 and Table 5. Visual comparison of accuracies is given in Figure 15.

As can be deduced from the figure, the 24 × 24 HOG descriptor performs significantly better for smaller dataset sizes (like 300/300). This is quite obvious, taking into account the difference in the size of space parameters. For 1200/1200 samples and above, both resolutions offer similar performance. The accuracy (measured by AUC) saturates at about 1200/1200 samples, so for this pattern there is little justification to use larger training sets. An attractive choice of parameters seems to be 24 × 24 HOG descriptor with 600/600 training samples. In this experiment this solution offered almost top accuracy accompanied by very quick training (only 49 s.) and detection (63 ms. per frame). The 24 × 24 HOG descriptor with 300/300 samples may also look attractive, but it more depends on the actual data selection (high standard deviation of results for different runs), and the performance is expected to differ more between consecutive runs.

It should be noted that the number of required samples strongly depends on the complexity of the pattern and its uniqueness, so it is tough to establish the value in general. Therefore this discussion should be treated rather as a proposition of some sane default values for the dataset size parameter in the system.

#### 3.2.6. VGG16 as 2-nd Stage Classifier

In this part of the experiments, the VGG16 network was utilized as the last stage classifier. After pre-detection by the cascade classifier, the results were fed to the VGG. The classifier, pre-trained on the ImageNet dataset, was immediately trained on-line on the set of gathered training samples. The number of training samples was set to 2400 for each class. There were evaluated four variants of the classifier: accepting images of resolution 32 × 32, 64 × 64, 128 × 128, and 224 × 224. The selection of a particular image size induced different numbers of layers. The training set of images was divided into training and validation using proportion 5:1. Only the fully-connected layers in the network were trained. The network was trained for a maximum of 6 epochs (for 224 × 224 resolution) or 10 epochs for smaller resolutions using Stochastic Gradient Descent or up to the moment when the validation error started to increase (using early-stopping principle). Practically in all cases, the network was able to train up to 100% of accuracy on the validation set.

The results obtained are given in the Table 6. The overall performance of the detector using VGG16 network can be regarded as acceptable, with the value of AUC greater than 0.7. It should be noted that the AUC value is quite similar for different network configurations, but the differences between them manifest in the *average precision-recall* measure. As could be expected, the full-width version, accepting images 224 × 224, where the output of the convolution part is 7 × 7 × 512 (as in the original paper), offers the best performance. The worst performance is offered by the network version accepting input 32 × 32, where the output of the convolution part is 1 × 1 × 512. The performance hit is probably due to getting rid of the information of feature location (the output is only 1 pixel wide). In such a configuration, the network is able to take into account the *presence* of features but not their position.

The full-width version of the network additionally has a prohibitive computational complexity of detection (even on a good GPU) consuming almost 1.5 s per each frame (compare it to classifier HOG+SVM classifier also given in the Table 6). The overall accuracy for VGG16 turns out to be worse than HOG+SVM. The VGG network suffers from the hidden overfitting problem (not manifesting itself in validation set error) due to incomplete training data. On the other hand, it seems that SVM+HOG classifier benefits more from the fully generic HOG feature extractor. The VGG16 network should perform better after some retraining of the specialized convolutional layer using enough quality data. Our unreported experiments show that the training data available in our problem is not sufficient for this purpose.

#### 3.2.7. Application of Faster-RCNN to Preprocessed Data

As the final experiment involving neural networks, we evaluated the Faster-RCNN [29] detector on our test sequence ’00012’ using as the training input the data collected and preprocessed using methods described in this paper. In order to prepare data acceptable by RCNN, augmented training images for hat pattern and their masks were superimposed on the negative training images in a regular grid-like fashion using masks for effective blending. The ROIs of input images were defined accordingly.

The original Faster-RCNN network was then re-trained with respect to the new data for 3000 training loops (which took 15 minutes in the Google Colab [47] environment). Then, the detection was performed, which took about 2 s per frame.

The Faster-RCNN network was able to achieve AUC = 0.67 and AVGPR = 0.56. The results are comparable to the ones obtained using VGG16 classifier and worse than the pair HOG+SVM.

#### 3.2.8. Detection of Various Patterns

**Figure 16:**
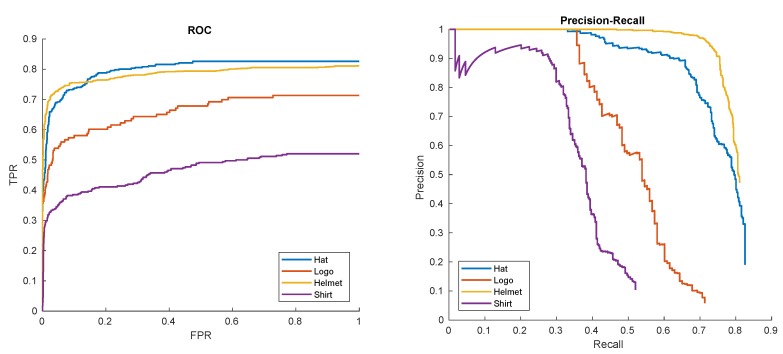
Receiver Operator Characteristic (ROC) and Precision-Recall (PR) curves of hat, logo, helmet, and shirt detections in ’00012’ sequence.

In the last of our preliminary experiments we evaluated how the detector handles different types of patterns. Therefore, the pattern logo was trained on a single training example with no mask, the pattern shirt was trained on a sequence of 30 samples without a mask and the pattern helmet was trained on 41 samples also without a mask using HOG+SVM for the second stage classifier. The results are given in Figure 16.

The relatively worse performance for the shirt pattern is mainly due to numerous occlusions. Even in the case of the ’shirt’ pattern we still have about 90% of successful hits for recall rates of 0.3. For best patterns, such as helmet, we have about 50% of positive examples with still 0 false positives!

In the course of the experiments, it was observed that motion blur (inherent or originating from de-interlacing) is the most destructive type of noise regarding both the training and detection phase. In addition, due to quite severe subsampling of the pattern (down to 24×24), the detector may suffer from problems in distinguishing between patterns differing only in small details. On the other hand, due to this property, the detector should well handle also small patterns—only slightly bigger than the nominal 24×24 pattern size.

### 3.3. Large-Scale Experiments

**Figure 17:**
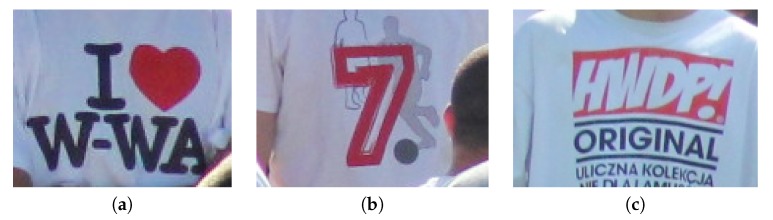
Three tested t-shirt logo patterns: (**a**) pattern P1, (**b**) pattern P2, (**c**) pattern P3.

Tests of the presented algorithm were conducted on a dataset containing 11 recordings, with nearly 30 thousand frames in total, with full HD resolution. Three patterns were created (Figure 17), and all sequences were carefully labeled by hand to create ground-truth data. All patterns were created based on a single frame (one positive sample). As training data, a high quality still picture was used, with resolution scaled down to full HD.

**Figure 18:**
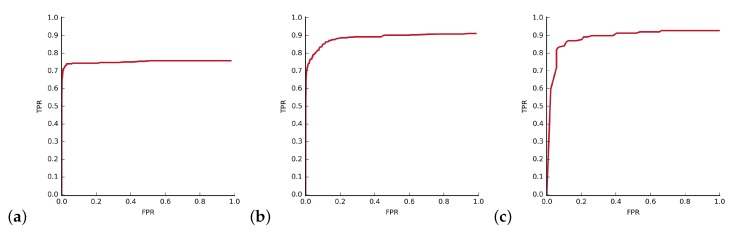
(**a**) ROC curve for the pattern P1. (**b**) Accumulated ROC curve for 5-elements sequence analysis. (**c**) ROC curve for cereal_1 object in desk_3 sequence.

Results of the experiments (ROC curve) for the selected pattern P1 are presented in Figure 18a. EER is similar for all patterns P1–P3, and is equal to 25.3%, 28.3%, and 28.0% for each pattern, respectively. Accumulated EER equals to 27.4%. Obtained results resemble those from smaller dataset. Even though the training sample and query images were obtained from different devices and had a different quality, the algorithm gave satisfactory results.

The final addition to the testing scenario was the utilization of short sequences. For every short time window, from all the results, only the one with the best response was taken as a final detection and passed to further processing. Accumulated results for the windows of length 5 is presented in Figure 18b. EER for them are, respectively: 27.4%, 15.1%, and 14.3%. It was observed that the longer the window the smaller is the quality gain.

From the point of view of a surveillance system operator, the system does not need to correctly annotate each and every frame in the sequence. In reality, what the operator expects is at least a single detection per continuous segment of appearances of the object. If the system can issue an alert, that the object is present in a single frame from such sub-sequence, the operator can perform further investigation manually.

To evaluate this, the experiments were performed concerning the detection of two selected patterns in seven different sequences. The detection was evaluated on per-segment and not per-frame basis. The ground (GT) for the movie sequence was composed of positive and negative segments. A positive segment was the continuous segment of positive samples; negative segment was a continuous segment of negative samples. Similar positive and negative segments were extracted from the detection result by applying hysteresis thresholds to the detector response. If the positively evaluated segment overlapped the true segment, it was considered ((H)it). In other case, it was considered ((M)iss). If the negatively evaluated segment overlapped the true positive segment, it was a ((S)kip).

Results are presented in Table 7. The Fragmentation describes the ratio of good Hits (multiple hits in a single segment are possible) to the number of segments in the ground truth data (GT). For each sequence, we had the information about the total number of times the target appears (number of continuous time segments, GT).

The most important conclusion here is that every continuous segment with the query pattern was hit at least once. The total fragmentation of 1.53 means, that the operator, on average, will be notified only slightly more often than he or she expects. Number of Misses, although not very small (0.42 of expected detections), is balanced by no Skips, which is the most important factor in the surveillance systems.

Additional experiments were conducted using one of the publicly available datasets. As our system relies on the masks, and one of the key concepts is to model the 3D object based on the small number of 2D views, we decided to use the Washington RGB-D dataset [48]. This dataset contains training sequences with a single object with labels (masks) and testing sequences with complex scenes containing multiple objects. In the experiments, the operator used the training sequence to pick the object of interest, and our system continued with the creation of the masks from the provided short sequence. Masks available in the dataset were used as a measure of the accuracy impact of automatic mask generation. After creating the model from a few images object was searched in the complex scenes. Figure 18c presents sample results obtained for the cereal_1 object in desk_3 sequence. The model was created using only 7 views of the object in this case, without using the GrabCut for mask creation.

## 4. Conclusions

In this paper, we presented a solution that can support the work of surveillance system operators. The system proved to positively address difficult task requirements concerning small training data sets, quick learning, and fast and reliable detection. An attractive training/detection speed and recognition rate trade-off was obtained by the application of a 2-layer cascade/SVM classifier. Additionally, a combination of cascade classifier and CNN was evaluated in the paper. The system proposed can learn from a single training sample but also can collect samples from short image sequences with only small user supervision in order to obtain rich training data. The addition of background subtraction and GrabCut for pattern generation made the process even more reliable. The performance of the system varies depending on the type and quality of training/test data, but we argue that, on average, results are satisfactory, and even not-the-best results provide sufficient information to be useful in practical surveillance scenarios.

## Figures and Tables

**Table 1:** Detection results with application of GrabCut.

	$c_{thr}=30$	$c_{thr}=130$	$c_{thr}=300$	$c_{thr}=600$
AUC avg.	0.76±0.02	0.77±0.01	0.78±0.63	0.76±0.07
AUC st.dev.	0.02	0.01	0.02	0.06
AVGPR avg.	0.69±0.03	0.73±0.01	0.73±0.02	0.72±0.07
AVGPR st.dev.	0.02	0.00	0.02	0.06

**Table 2:** Detection results without application of GrabCut.

	$c_{thr}=30$	$c_{thr}=130$	$c_{thr}=300$	$c_{thr}=600$
AUC avg.	0.73±0.08	0.74±0.05	0.71±0.04	0.64±0.07
AUC st.dev.	0.07	0.04	0.04	0.06
AVGPR avg.	0.65±0.11	0.66±0.05	0.62±0.08	0.51±0.08
AVGPR st.dev.	0.10	0.05	0.07	0.07

**Table 3:** Comparison of detection results using Background Subtraction + GrabCut (BS + GC), pure Background Subtraction (BS), and SiamMask [45] method for collecting training samples.

	BS + GC: $c_{thr}=300$	BS: $c_{thr}=130$	SiamMask
AUC avg.	0.78±0.03	0.74±0.05	0.66±0.03
AUC st.dev.	0.02	0.04	0.03
AVGPR avg.	0.73±0.02	0.66±0.05	0.54±0.05
AVGPR st.dev.	0.02	0.05	0.04

**Table 4:** Detector performance for 24 × 24 HOG features and different number of training samples per class.

	HOG24(300)	HOG24(600)	HOG24(1200)	HOG24(2400)
AUC avg.	0.77±0.05	0.77±0.01	0.80±0.01	0.81±0.01
AUC st.dev.	0.04	0.01	0.01	0.01
AVGPR avg.	0.73±0.06	0.73±0.01	0.75±0.01	0.77±0.02
AVGPR st.dev.	0.05	0.00	0.01	0.01
training (sec.)	33	49	92	170
detection(msec/fr.)	58	63	65	63

**Table 5:** Detector performance for 32 × 32 HOG features and different number of training samples per class.

	HOG32(300)	HOG32(600)	HOG32(1200)	HOG32(2400)
AUC avg.	0.69±0.06	0.73±0.05	0.80±0.02	0.80±0.01
AUC st.dev.	0.05	0.04	0.02	0.01
AVGPR avg.	0.59±0.12	0.66±0.05	0.76±0.02	0.77±0.00
AVGPR st.dev.	0.10	0.04	0.02	0.00
training (sec.)	44	68	171	353
detection (msec/fr.)	70	77	107	100

**Table 6:** Performance of VGG16 network as a second stage classifier. Last column contains HOG results for reference.

	VGG16(32)	VGG16(64)	VGG16(128)	VGG16(224)	HOG(2400)
AUC avg.	0.69±0.05	0.72±0.01	0.72±0.02	0.72±0.05	0.80±0.01
AUC st.dev.	0.06	0.01	0.01	0.05	0.01
AVGPR avg.	0.38±0.07	0.45±0.03	0.54±0.04	0.61±0.06	0.77±0.00
AVGPR st.dev.	0.08	0.03	0.03	0.06	0.00
training (sec.)	60	74	140	223	353
detection(msec./fr.)	118	225	466	1457	100

**Table 7:** Continuous sequence detection results.

Sequence	GT	Hit	Skip	Miss	Fragmentation
146	1	3	0	0	3
154	3	3	0	2	1
162 (A)	1	1	0	0	1
162 (B)	8	14	0	3	1.75
163	2	2	0	0	1
164	3	5	0	0	1.67
168	1	1	0	3	1
total	19	29	0	8	1.53

## Abbreviations

The following abbreviations are used in this manuscript:

AUC	Area Under Curve (ROC)
AVGPR	Average Precision-Recall
CC	Cascade Classifier
CNN	Convolutional Neural Network
CSK	Circulant Structure of Kernels
EER	Equal Error Rate
FPR	False Positive Rate
GPU	Graphic Processing Unit
HD	High Definition
HOG	Histogram of Oriented Gradients
PR	Precision-Recal (curve)
P-REACT	Petty cRiminality diminution through sEarch and Analysis
	in multi-source video Capturing and archiving plaTform
RBF	Radial Basis Function
RGB-D	Red Green Blue-Depth
ROC	Receiver Operator Characteristics
ROI	Region of Interest
RPCA	Robust Principal Component Analysis
SIFT	Scale Invariant Feature Transform
SURF	Speeded-Up Robust Features
SVM	Support Vector Machine
TPR	True Positive Rate
